# Correlative light-electron microscopy methods to characterize the ultrastructural features of the replicative and dormant liver stages of *Plasmodium* parasites

**DOI:** 10.1186/s12936-024-04862-w

**Published:** 2024-02-21

**Authors:** Gabriel Mitchell, Laura Torres, Matthew E. Fishbaugher, Melanie Lam, Vorada Chuenchob, Reena Zalpuri, Shreya Ramasubban, Caitlin N. Baxter, Erika L. Flannery, Anke Harupa, Sebastian A. Mikolajczak, Danielle M. Jorgens

**Affiliations:** 1 Open Innovation at Global Health Disease Area, Biomedical Research, Novartis, Emeryville, CA USA; 2 Global Health Disease Area, Biomedical Research, Novartis, Emeryville, CA USA; 3 Electron Microscope Laboratory, University of California, Berkeley, CA USA

**Keywords:** Plasmodium berghei, Plasmodium cynomolgi, CLEM, TEM, Schizonts, Hypnozoites, Relapsing malaria, Transmission electron microscopy, Hepatocytes, Mitochondria

## Abstract

**Background:**

The infection of the liver by *Plasmodium* parasites is an obligatory step leading to malaria disease. Following hepatocyte invasion, parasites differentiate into replicative liver stage schizonts and, in the case of *Plasmodium* species causing relapsing malaria, into hypnozoites that can lie dormant for extended periods of time before activating. The liver stages of *Plasmodium* remain elusive because of technical challenges, including low infection rate. This has been hindering experimentations with well-established technologies, such as electron microscopy. A deeper understanding of hypnozoite biology could prove essential in the development of radical cure therapeutics against malaria.

**Results:**

The liver stages of the rodent parasite *Plasmodium berghei*, causing non-relapsing malaria, and the simian parasite *Plasmodium cynomolgi*, causing relapsing malaria, were characterized in human Huh7 cells or primary non-human primate hepatocytes using Correlative Light-Electron Microscopy (CLEM). Specifically, CLEM approaches that rely on GFP-expressing parasites (GFP-CLEM) or on an immunofluorescence assay (IFA-CLEM) were used for imaging liver stages. The results from *P. berghei* showed that host and parasite organelles can be identified and imaged at high resolution using both CLEM approaches. While IFA-CLEM was associated with more pronounced extraction of cellular content, samples’ features were generally well preserved. Using IFA-CLEM, a collection of micrographs was acquired for *P. cynomolgi* liver stage schizonts and hypnozoites, demonstrating the potential of this approach for characterizing the liver stages of *Plasmodium* species causing relapsing malaria.

**Conclusions:**

A CLEM approach that does not rely on parasites expressing genetically encoded tags was developed, therefore suitable for imaging the liver stages of *Plasmodium* species that lack established protocols to perform genetic engineering. This study also provides a dataset that characterizes the ultrastructural features of liver stage schizonts and hypnozoites from the simian parasite species *P. cynomolgi*.

**Graphical Abstract:**

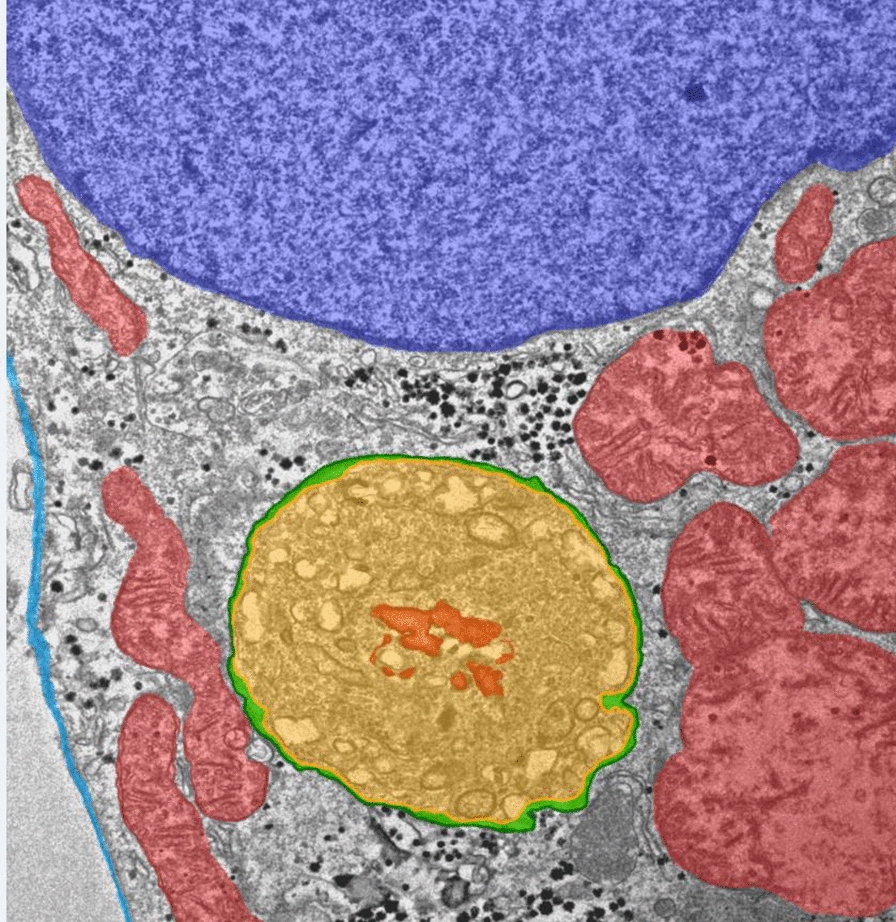

**Supplementary Information:**

The online version contains supplementary material available at 10.1186/s12936-024-04862-w.

## Background

Malaria is a highly impactful disease that globally affects hundreds of millions of people each year [1]. Complete eradication of malaria must encompass the elimination *of Plasmodium vivax* alongside *Plasmodium falciparum* and other less dominant species, as *P. vivax*’s ability to remain dormant in the liver causes clinical relapses and foster dissemination. However, the unique biology of *P. vivax* makes it challenging to study, impeding the development of effective interventions [2].

Infection of the mammalian host by *Plasmodium* parasites is initiated by a mosquito bite, which releases sporozoites into the bloodstream, ultimately allowing them to enter the liver and invade hepatocytes. Once inside the host cell, sporozoites further differentiate and develop within a parasitophorous vacuole (PV), delineated by a PV membrane (PVM) that constitutes the primary host–pathogen interface. Parasites then replicate their genome thousands of times through schizogony prior to the formation of merozoites that are released in the bloodstream to initiate the symptomatic phase of the infection [3]. For *Plasmodium* species causing relapsing malaria (e.g., *P. vivax* and *Plasmodium cynomolgi*), dormant liver stages (i.e., hypnozoites) can also form before schizogony and activate days or months after the initial infection, leading to clinical relapses. Novel therapeutics against hypnozoites are needed for malaria eradication as hypnozoites are highly tolerant to most anti-malarials [4]. Currently, the only available drugs active against hypnozoites have adverse effects on patients with glucose-6-phosphate dehydrogenase (G6PD) deficiency [2].

Most of the knowledge on the liver stage of *Plasmodium* parasites comes from rodent parasite species (e.g., *Plasmodium berghei* and *Plasmodium yoelii*). These species offer a good genetic tractability and develop into liver stage schizonts, not only in primary hepatocytes but also in several immortalized cell lines [3]. Nevertheless, rodent *Plasmodium* parasites are unsuitable models for studying relapsing malaria as they do not form hypnozoites.

While some of the challenges posed by the unique biology of *P. viva*x can be overcome by using the simian parasite species *P. cynomolgi*, including the lack of continuous culturing methods [5, 6], studying hypnozoite biology remains difficult due to many technical hurdles. As of now, there have been no published reports on the genetic engineering of *P. vivax* and creating transgenic parasites in *P. cynomolgi* can be extremely labour-intensive and the methodology is still in its infancy [7–9]. Moreover, conducting research on the liver stages of *P. vivax* and *P. cynomolgi* requires access to scarce resources, including mosquitoes that have fed on infected human or non-human primate (NHP) blood as well as primary hepatocytes [2, 10]. This is in addition to the other outstanding challenges in studying liver stage biology such as the low infection rate.

Although the ultrastructure of liver stage schizonts of *Plasmodium* species causing non-relapsing malaria has been previously resolved using transmission electron microscopy (TEM) [11], there is only a limited number of studies utilizing more advanced electron microscopy (EM) techniques to characterize this stage [12, 13]. EM data on the liver stages of *Plasmodium* species causing relapsing malaria is sparse, with only brief reports on *P. cynomolgi* [14] and *P. vivax* [15] schizonts. No EM data characterizing hypnozoites has been reported in the literature.

The low infection rate during the liver phase of infection and the diminutive size of hypnozoites pose a significant challenge to their localization and identification using conventional EM. These obstacles can be overcome using correlative light-electron microscopy (CLEM), a powerful approach that allows tracking of rare biological events, initially using fluorescence microscopy, followed by the characterization of their ultrastructure at high-resolution with EM [16]. Importantly, CLEM has already been utilized to characterize *P. berghei* liver stages [13], suggesting its applicability to study the liver stages of other *Plasmodium* species. Acquiring thorough ultrastructural datasets using CLEM will contribute to addressing the significant gaps in the fundamental understanding of the liver stages of *Plasmodium* species causing relapsing malaria.

This study aimed to optimize CLEM protocols for the liver stages of *Plasmodium* species causing non-relapsing and relapsing malaria. Specifically, CLEM protocols relying on using parasites expressing GFP (GFP-CLEM) or on an immunofluorescence assay (IFA-CLEM) for detecting *Plasmodium* liver stages were first optimized and compared using *P. berghei*. The IFA-CLEM approach was then used to conduct experiments with genetically-unmodified *P. cynomolgi*, resulting in a set of micrographs characterizing liver stage schizonts and hypnozoites. Overall, this study demonstrates that CLEM is an effective tool for gaining insights into the ultrastructure of the sparsely distributed *Plasmodium* liver stages.

## Methods

See point-by-point protocols to perform GFP-CLEM and IFA-CLEM on *Plasmodium* liver stages (Additional file 1) and the list of samples analysed during this study (Additional file 2).

## Cell lines and primary hepatocytes

The human hepatoma Huh7 cell line was grown in Dulbecco’s modified Eagle’s medium (DMEM) with high glucose (Gibco) supplemented with 10% fetal bovine serum (FBS; Corning), 100 U/mL of penicillin–streptomycin (Gibco) and 1% GlutaMAX^™^ (Gibco) (hereafter referred to as Huh7 medium). Primary rhesus macaque or cynomolgus hepatocytes (BioIVT; lots LZX, HTV or CWP) were maintained in INVITRO CP medium (BioIVT) supplemented with 1% of a Penicillin–Streptomycin-Neomycin (PSN) mixture (ThermoFisher, Catalog no.15640055) (hereafter referred to as Hep medium), unless otherwise specified.

## Infection of Huh7 cells with *P. berghei*

Dishes containing gridded coverslips (MatTek, P35G-1.5–14-CGRD) were incubated for 1 h at room temperature (RT) in 50 μg/mL of poly-D-lysine (PDL; Gibco), rinsed thrice in Dulbecco's Phosphate Buffered Saline (D-PBS; Gibco) and dried, with lids off, in a biosafety cabinet for 2 h. Huh7 cells were seeded at a density of 700,000 cells per PDL-coated dish one day prior to the infection. *Anopheles stephensi* mosquitoes infected with *P. berghei* strain ANKA GFP-LUC_CON_ [17] were produced by the SporoCore (University of Georgia) and sporozoites were isolated by microdissection of salivary glands, as previously described with modifications [18, 19]. Sporozoites were diluted in Roswell Park Memorial Institute (RPMI; Gibco) 1640 medium supplemented with 20% FBS and Huh7 were spin-infected with 500,000 sporozoites per dish at 330 × g for 3 min, with low acceleration and break. Cells were then incubated for 2 h at 37 °C and 5% CO_2_, cell culture medium was changed for Huh7 medium and infected cells were further incubated for 2 days at 37 °C and 5% CO_2_.

## Infection of primary hepatocytes with *P. cynomolgi*

Dishes containing gridded coverslips (MatTek) were incubated for 2 h at RT in 0.02 N acetic acid containing 50 μg/mL of rat tail collagen I (purchased from Corning or Enzo), rinsed thrice with D-PBS and air-dried, with lids off, in a biosafety cabinet for 2 h. Two days before the infection, 2 million hepatocytes were seeded in each collagen-coated dish and cell monolayers were monitored at least every other day using light microscopy. *Anopheles dirus* mosquitoes infected with the B strain of *P. cynomolgi* were produced by the Pathogen and Vector Interaction Section (PVIS) of the Department of Entomology at the Armed Forces Research Institute of Medical Sciences (AFRIMS; Bangkok, Thailand) under the IACUC approved animal use protocol PN22-10. *Anopheles dirus* mosquitoes were reared and maintained in the insectary of the Department of Entomology (AFRIMS; Bangkok, Thailand) following a method previously described [20]. Sporozoites were isolated from salivary glands by microdissection, as previously described with modifications [18, 19, 21]. Sporozoites were diluted in Hep medium and primary hepatocytes were spin-infected with 0.5–1 million sporozoites per dish at 200 × g for 5 min. Cells were then incubated for 2 h at 37 °C and 5% CO_2_ and the cell culture medium was changed for fresh Hep medium. The next day and then every 2–3 days, the Hep medium was changed for INVITRO CP medium supplemented with 5% of a PSN mixture (ThermoFisher) and cells were incubated until 7 dpi.

## Light and fluorescent imaging for GFP-CLEM

*Plasmodium berghei*-infected Huh7 cells were incubated at 37 °C and 5% CO_2_ for 30 min in FluoroBrite DMEM medium (Invitrogen) supplemented with 10% fetal bovine serum (FBS; Corning), 100 U/mL of penicillin–streptomycin (Corning), 1% GlutaMAX^™^ (Gibco) (hereafter referred to as Imaging medium) and 10 μg/mL Hoechst (ThermoFisher). Cells were then washed once with Imaging medium and fiducial markers were generated by scraping off linear patterns of cells from the monolayers. Fresh Imaging medium was then added to cell cultures and imaging was performed at RT and ambient air using a Nikon TI-E microscope. To collect light and fluorescent microscopy data facilitating the tracking of region of interests (ROI), images were taken at a range of magnifications, minimally using 4 × , 10 × , 20 × and 40 × objectives. Only 2–3 ROIs per sample were characterized to limit imaging time. Once imaged, samples were washed twice with D-PBS, incubated for 15 min at RT in D-PBS containing 2% paraformaldehyde (EMS) and 2% glutaraldehyde (EMS) and kept at 4 °C in the fixative solution and protected from light, until processing for CLEM.

## Immunostaining protocol and light and fluorescent imaging for IFA-CLEM

Samples were incubated in D-PBS containing 4% paraformaldehyde for 15 min at RT, washed twice in D-PBS, and kept at 4 °C in D-PBS until further processing. Samples were then incubated in 0.05% Triton X-100 (Sigma-Aldrich) D-PBS for 15 min, washed twice in D-PBS and incubated for 30 min in 2% bovine serum albumin (BSA; Sigma-Aldrich) D-PBS at RT. Samples were then incubated with primary antibodies (*P. berghei*, αUIS4_*Pb*_ goat IgG (SICGEN; 1:250); *P. cynomolgi*, αUIS4_*Pc*_ human IgG (1:2000)) for 3 h in 2% BSA D-PBS, washed 4 times in D-PBS and incubated with secondary antibodies (Alexa Fluor 568 donkey anti-goat IgG or Alexa Fluor 488 goat anti-human IgG (Invitrogen; 1:1000)) for 90 min in 2% BSA D-PBS containing 2 μg/mL Hoechst at RT. The αUIS4_*Pc*_ human IgG was produced in-house and is a mouse-derived antibody inserted into the human antibody backbone [19]. Samples were then washed twice in D-PBS and kept at 4 °C in the dark until imaging. Fiducial markers were generated by scraping off linear patterns of cells from the monolayers, samples were washed twice in D-PBS and images were acquired for multiple ROIs using a range of different magnifications and Nikon TI-E or Zeiss LSM 980 microscopes. Once imaged, samples were incubated for 15 min at RT in D-PBS containing 2% paraformaldehyde and 2% glutaraldehyde and kept at 4 °C, in the dark and in the fixative solution, until processing for CLEM.

## Preparation of samples for CLEM and TEM imaging

Samples were rinsed (3 × ; 5 min, RT) in PBS, pH 7.4, and immersed in 1% osmium tetroxide with 1.6% potassium ferricyanide in PBS for 30 min. Samples were rinsed (3 × ; 5 min, RT) in buffer and then briefly in distilled water (1 × ; 5 min, RT). Samples were then subjected to an ascending ethanol gradient (7 min; 35%, 50%, 70%, 80%, 90%) followed by pure ethanol (3 × ; 5 min, RT). Samples were progressively infiltrated while rocking with Epon resin (EMS) and polymerized at 60 °C for 12–18 h. Care was taken to ensure only a thin amount of resin remained within the glass bottom dishes, which enable the best possible chance for separation of the glass coverslip. Following polymerization, the glass coverslips were removed using ultra-thin Personna razor blades (EMS) and liquid nitrogen exposure, as needed. The ROIs, identified via the gridded alpha-numerical labelling, were carefully removed, and mounted with cyanoacrylate glue for sectioning on a blank block. Serial thin sections (80–90 nm) were cut using a Leica UC6 ultramicrotome (Leica) from the surface of the block (corresponding to the bottom of the cell layer) until approximately 20 μm to ensure complete capture of the cell volumes. Section-ribbons were then collected sequentially onto formvar-coated slot grids. During the serial sectioning process, a few thicker sections (250–350 nm) were collected onto glass slides and stained with toluidine blue to track the cells and nuclei of the ROIs and serve as a bridge from the fluorescence data into the TEM imaging. The TEM grids were post-stained with 2% uranyl acetate followed by Reynold’s lead citrate, for 5 min each. The sections were imaged using a Tecnai 12 120 kV TEM (FEI) and data were recorded using a Gatan Rio 16 CMOS with Gatan Microscopy Suite software (Gatan).

## Results

### Imaging of *P. berghei* liver stages using GFP-CLEM

**Fig. 1:**
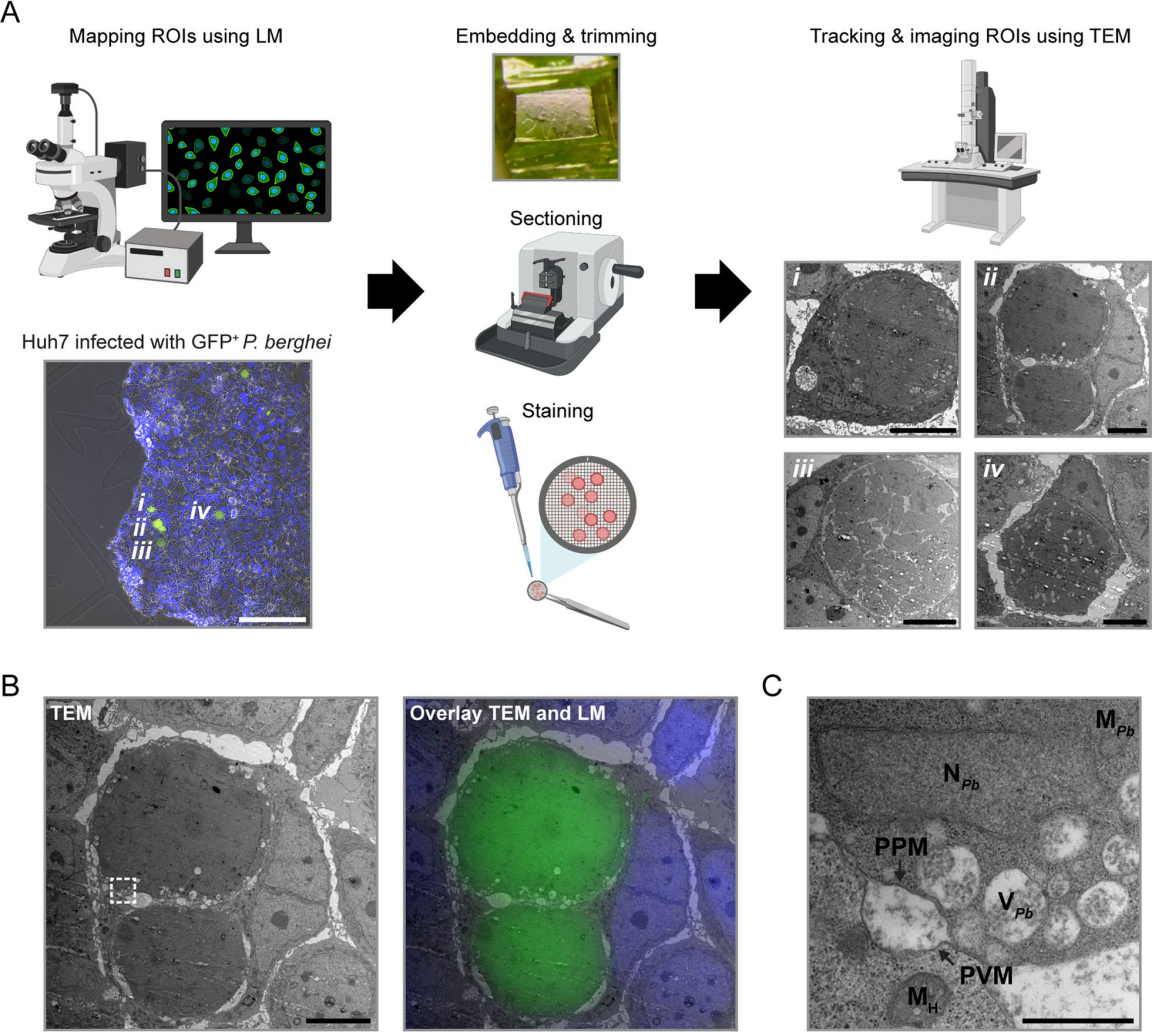
Imaging of *P. berghei* liver stages using GFP-CLEM. **A** Illustration outlining the protocol to perform CLEM of *P. berghei* liver stages expressing GFP. Huh7 cells were seeded on gridded coverslips presenting alphanumerical coordinates and infected with *P. berghei* sporozoites. Maps of cells infected with 2-day old liver stages were acquired using GFP (green) and Hoechst (a nucleic acid stain, blue) and light and fluorescence microscopy (LM). Samples were then processed for TEM imaging, which includes embedding samples in resin, trimming resin blocks around ROIs, preparing ultra-thin sections using the microtome and staining with contrasting reagents. Samples were then imaged using TEM and ROIs were located by correlating patterns of host nuclei and liver stages. An example shows a LM map (bottom left) and low-magnification TEM micrographs (bottom right) for 4 GFP^+^ liver stages (*i*, *ii*, *iii* and *iv*). Scale bars are 200 μm and 10 μm for the LM and TEM micrographs, respectively. Drawings were created with BioRender.com. **B** The overlay of micrographs exemplifies how data from LM and TEM are correlated and used to re-localize ROIs. Scale bar is 10 μm. **C** Higher-magnification TEM micrographs showing the hepatocyte-parasite interface, and a host cell mitochondrion (M_H_), a *P. berghei* mitochondrion (M_*Pb*_), the parasitophorous vacuole membrane (PVM), the parasite plasma membrane (PPM), *P. berghei* vacuoles (V_*Pb*_) and a *P. berghei* nucleus (N_*Pb*_). Scale bar is 1 μm. The inset in (**B**) defined by a box with a white dotted border shows the area selected for the zoom-in micrograph presented in (**C**)

An approach referred to as GFP-CLEM (Fig. 1) was first optimized using a transgenic line of *P. berghei* that constitutively expresses cytosolic GFP [17], similarly to a method previously described [13]. More specifically, Huh7 cells were seeded on gridded coverslips of glass-bottom dishes with an alphanumeric pattern and infected with sporozoites. Parasites were allowed to develop into intracellular liver stages for about 2 days prior to imaging using light and fluorescence microscopy. Samples were then further processed, which include trimming resin blocks around ROIs and screening for ROIs in toluidine blue-stained sections using light microscopy (LM), and imaged using TEM. Correlating the host nuclei pattern observed with fluorescence microscopy and TEM allowed precise tracking and high-resolution imaging of liver stages. The process is outlined in Fig. 1A and 1B. Liver stages lack synchronization at 2 days post-infection (dpi) and different developmental stages were observed, including mature schizonts (e.g., Fig. 1A, Micrograph *ii*) and schizonts undergoing segmentation (e.g., Fig. 1A, Micrograph *iii*). Importantly, known ultrastructural features were identified in both the host (e.g., nuclei (Fig. 1B) and mitochondria (Fig. 1C)) and the parasites (e.g., nuclei, mitochondria, PVM, PPM and vacuoles (Fig. 1C)), confirming that GFP-CLEM can be used to image *P. berghei* liver stages.

## Imaging of *P. berghei* liver stages using IFA-CLEM

**Fig. 2:**
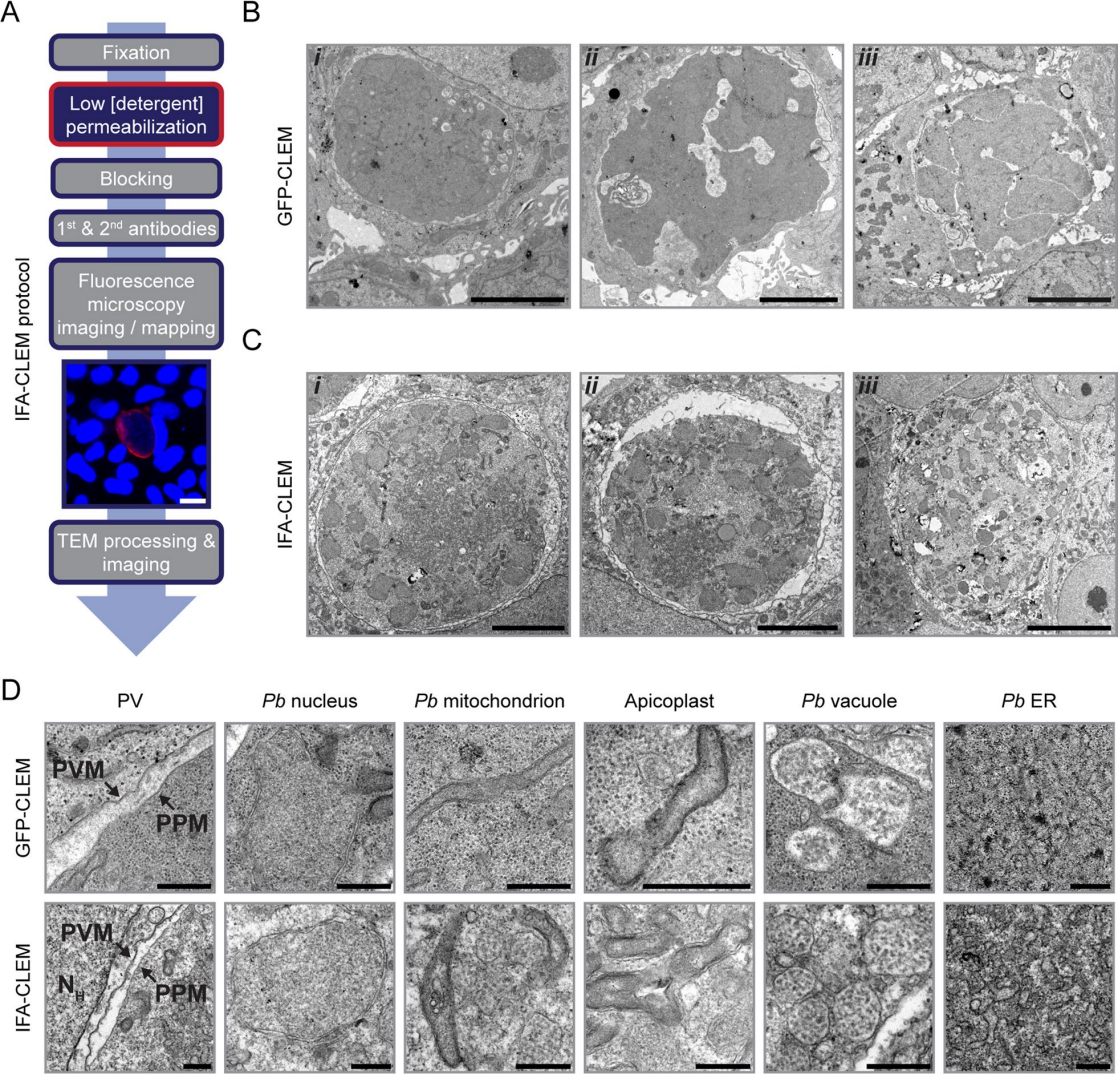
Imaging of *P. berghei* liver stages using IFA-CLEM. **A** Diagram outlining the IFA protocol used to perform IFA-CLEM. A representative image of a 2-day old *P. berghei* liver stage in Huh7 cells stained for the PVM protein UIS4 (red) and Hoechst (blue) is shown. Scale bar is 20 μm. Low-magnification TEM micrographs for liver stages imaged with GFP-CLEM (**B**) and IFA-CLEM (**C**). Scale bars are 5 μm (B-*i*, B-*ii*, C-*i* and C-*ii*) or 10 μm (B-*iii* and C-*iii*). **D** Higher-magnification GFP-CLEM (top row) and IFA-CLEM (bottom row) micrographs showing the parasitophorous vacuole (PV) space, *P. berghei* (*Pb*) nuclei, the *P. berghei* mitochondrial network, apicoplasts, *P. berghei* vacuoles and the *P. berghei* endoplasmic reticulum (ER). N_H_, host nucleus; PVM, parasitophorous vacuole membrane; PPM, parasite plasma membrane. Scale bars are 500 nm

The low genetic tractability of *Plasmodium* species causing relapsing malaria hinders the characterization of their liver stages using CLEM approaches that rely on genetically encoded tags. To address this challenge, a CLEM protocol based on an immunofluorescent assay (referred to as IFA-CLEM) was developed using Huh7 host cells and *P. berghei* (Fig. 2). The IFA protocol involved a short permeabilization step with reduced detergent concentration to minimize membrane damage and artifact occurrence during TEM processing [16]. The simplified protocol of IFA-CLEM is outlined on Fig. 2A. Interestingly, the PVM protein UIS4 (Fig. 2A), but not the cytosolic parasite protein HSP70, could be detected using this IFA protocol, confirming a limited permeabilization of samples. The comparison of *P. berghei* liver stage images acquired with GFP-CLEM (Fig. 2B) and IFA-CLEM (Fig. 2C) validated the latter approach. While IFA-CLEM produced samples less opaque to electrons with more extraction of intracellular contents (Fig. 2C), *P. berghei* ultrastructural features and organelles were well defined and could be identified, including the PV, nuclei, the mitochondria network, the apicoplast, the endoplasmic reticulum (ER) and vacuoles (Fig. 2D, Additional file 3). The parasite’s nuclei and ER were easier to identify using IFA-CLEM than GFP-CLEM (Fig. 2D and Additional File 3). The ER had the expected unusual architecture, as previously reported [12]. Unknown parasite protuberances associated with the apicoplast that extend into the host cell were observed using both CLEM approaches (Additional File 4). It remains to be determined if these protuberances are involved in host-parasite interactions or in the development of liver stage parasites (e.g., by contributing to the formation of the PV). Overall, these results demonstrate the utility of IFA-CLEM for studying the ultrastructure of the liver stages of *Plasmodium* parasites.

## Imaging of *P. cynomolgi* liver stage schizonts using IFA-CLEM

**Fig. 3:**
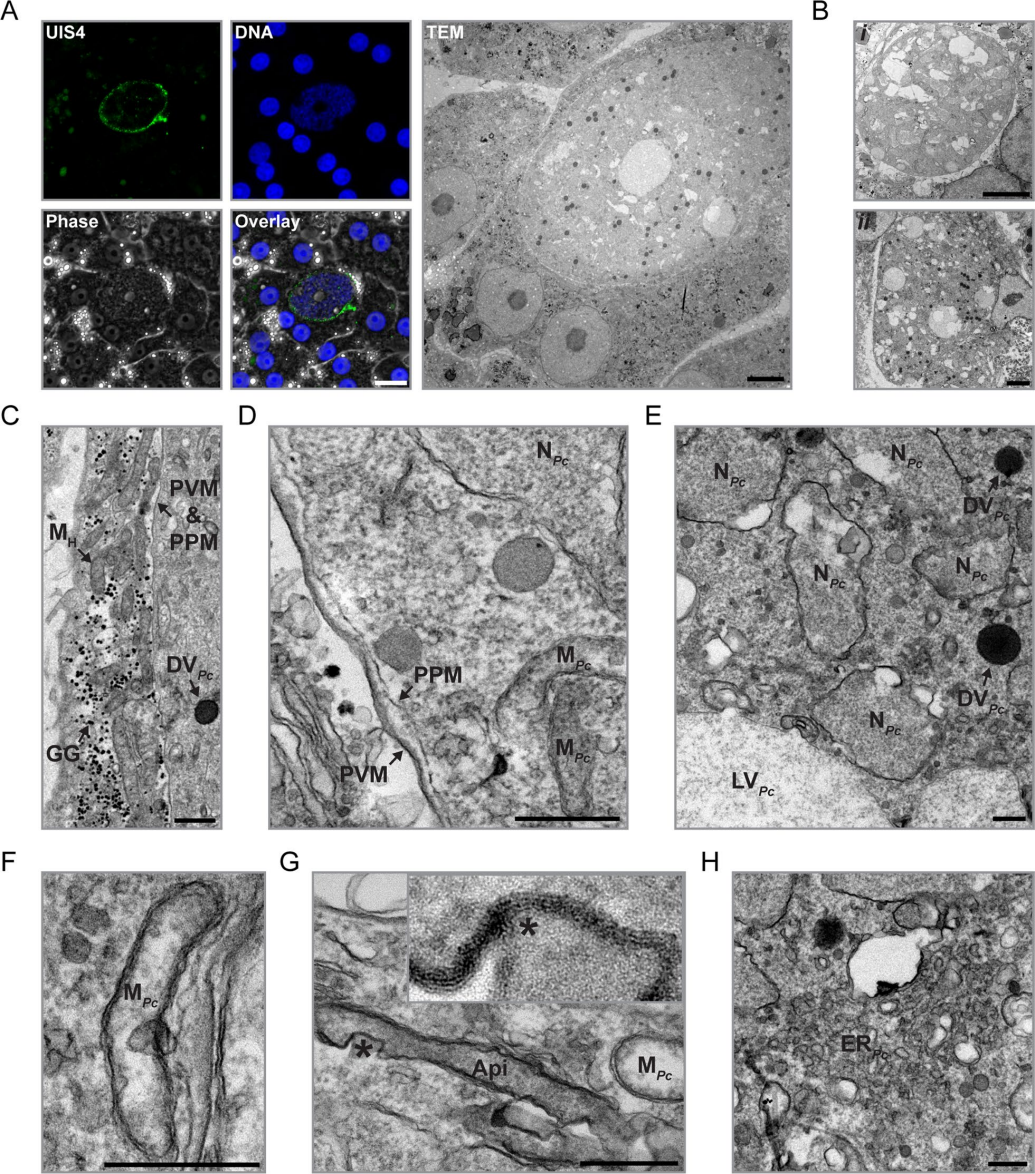
Imaging of *P. cynomolgi* liver stage schizonts using IFA-CLEM. **A** Micrographs showing a *P. cynomolgi* schizont at 7 dpi in a primary NHP hepatocyte stained for UIS4 (green) and nucleic acids (Hoechst, blue) and imaged with fluorescence and phase contrast microscopy. Scale bar is 20 μm. The same schizont is also represented on a TEM micrograph obtained using IFA-CLEM. Scale bar is 5 μm. **B** TEM micrographs of two other *P. cynomolgi* schizonts at 7 dpi (*i* and *ii*). Scale bars are 5 μm. **C**–**H** TEM micrographs showcasing the ultrastructural features of *P. cynomolgi* liver stage schizonts, including an apicoplast (Api), host glycogen granules (GG), host mitochondria (M_H_), dense/electron-opaque *P. cynomolgi* vacuoles (DV_*Pc*_), large *P. cynomolgi* vacuoles (LV_*Pc*_), the *P. cynomolgi* endoplasmic reticulum (ER_*Pc*_), the *P. cynomolgi* mitochondrial network (M_*Pc*_), *P. cynomolgi* nuclei (N_*Pc*_) and the parasitophorous vacuole (PV) in hepatocytes infected with *P. cynomolgi* schizonts at 7 dpi. Note the host mitochondria positioned in proximity to the PVM in (**C**). PVM, parasitophorous vacuole membrane; PPM, parasite plasma membrane. Scale bars are 1 μm (**C**) or 500 nm **D**–**H**

To investigate the applicability of IFA-CLEM in studying the liver stages of *Plasmodium* species causing relapsing malaria, primary NHP hepatocytes infected with *P. cynomolgi* sporozoites were examined at 7 dpi (Fig. 3), a time point at which schizonts and hypnozoites can be differentiated based on size and number of DNA punctae [21]. As observed for *P. berghei* staining (Fig. 2), the IFA-CLEM protocol effectively stained the PVM protein UIS4, allowing for the localization of *P. cynomolgi* schizonts (Fig. 3A-B). Upon further examination at higher magnification, it was observed that the host mitochondrial network was frequently found near the PVM in *P. cynomolgi* schizonts (Fig. 3C and Additional File 5). At least for the time points imaged in this study, the PV space of *P. cynomolgi* schizonts appeared narrower in comparison to *P. berghei* (Figs. 2 and 3D). IFA-CLEM was effective in allowing the identification of various organelles in *P. cynomolgi* schizonts, including nuclei (Fig. 3E), the mitochondrial network (Fig. 3D, F, and G), the apicoplast with its 4 membrane layers (Fig. 3G) [22], and the ER (Fig. 3H). Unlike the 2-day old *P. berghei* liver stages, *P. cynomolgi* schizonts were found to have both large electron-translucent and electron-opaque (or dense) vacuoles at 7 dpi (Fig. 3C and E). The presence of dense vacuoles in *P. cynomolgi* liver stage schizonts was previously reported [14]. It is also noteworthy that regions of nuclei were electron-translucent in *P. cynomolgi* liver stages (Fig. 3E), which may be an artifact caused by the IFA-CLEM protocol or have a biological explanation. The results of this section confirm that IFA-CLEM can be utilized to characterize *P. cynomolgi* schizonts.

## Imaging of *P. cynomolgi* hypnozoites using IFA-CLEM

**Fig. 4:**
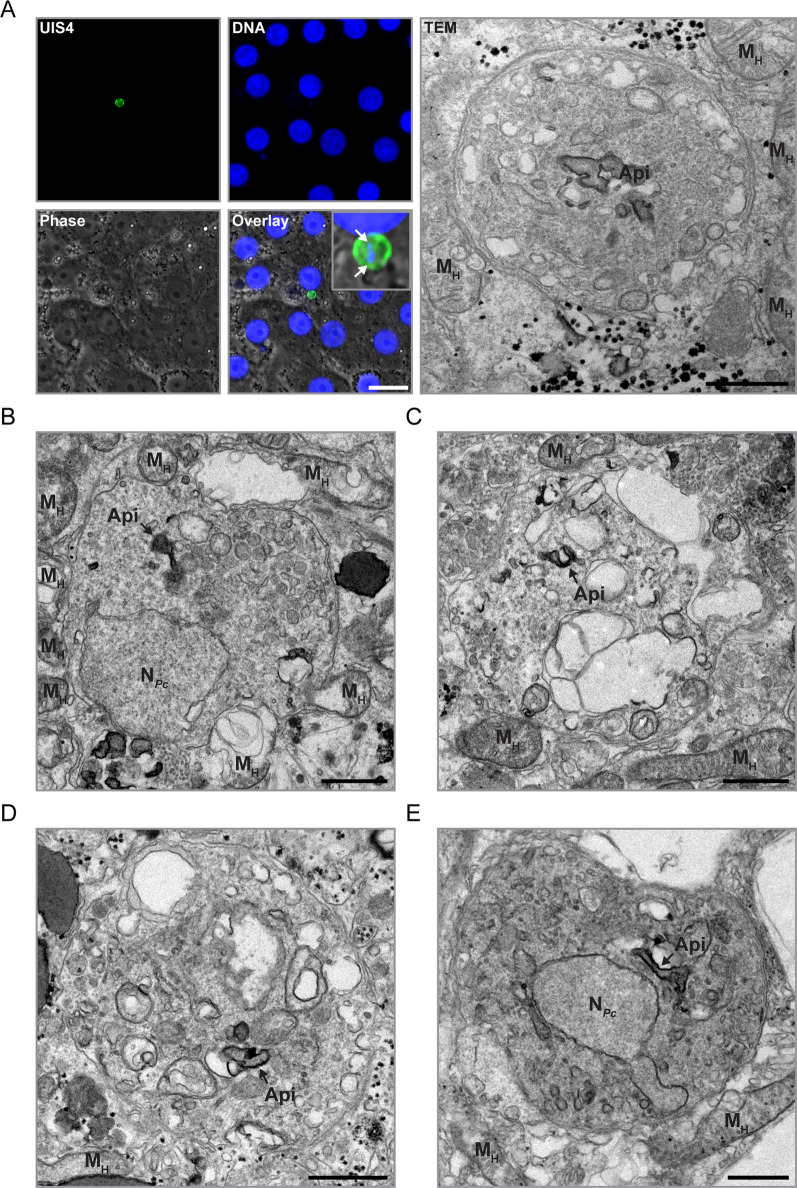
Imaging of *P. cynomolgi* hypnozoites using IFA-CLEM. **A** Micrographs showing a *P. cynomolgi* hypnozoite at 7 dpi in a primary NHP hepatocyte stained for UIS4 (green) and nucleic acids (Hoechst, blue) and imaged with fluorescence and phase contrast microscopy. Scale bar is 20 μm. The white arrows on the zoom-in micrograph (top right of overlay) show two DNA punctae in the hypnozoite. The same hypnozoite is also represented on a TEM micrograph obtained using IFA-CLEM. Scale bar is 1 μm. **B**–**E** Low-magnification TEM micrographs of additional hypnozoites at 7 dpi. Note the host mitochondria (M_H_) positioned in proximity to the PVM of hypnozoites. N_*Pc*_, *P. cynomolgi* nuclei; Api, apicoplast. Scale bars are 1 μm

**Fig. 5:**
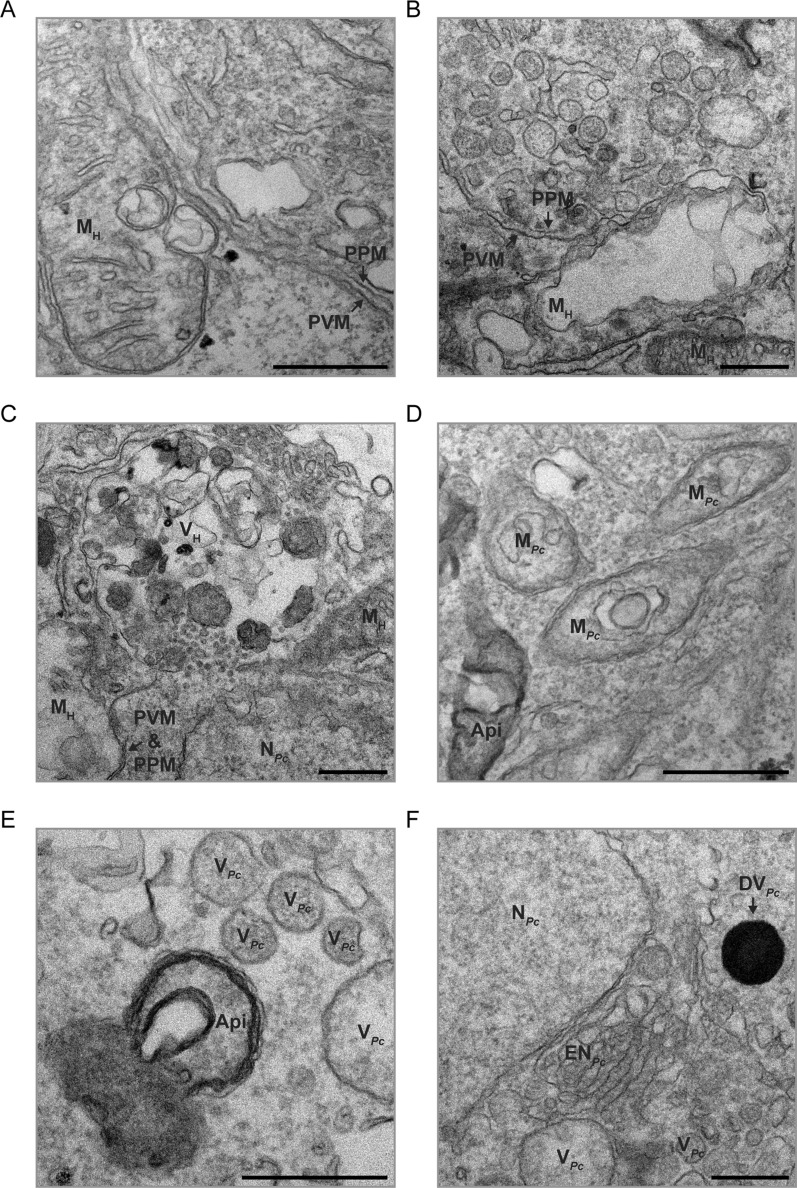
Ultrastructure of *P. cynomolgi* hypnozoites. **A**–**F** TEM micrographs showcasing the apicoplast (Api), host mitochondria (M_H_), a host vacuole (V_H_), a dense/electron-opaque *P. cynomolgi* vacuole (DV_*Pc*_), electron-translucent *P. cynomolgi* vacuoles (V_*Pc*_), the *P. cynomolgi* endomembrane network (EN_*Pc*_), the *P. cynomolgi* mitochondrial network (M_*Pc*_), *P. cynomolgi* nuclei (N_*Pc*_) and the parasitophorous vacuole (PV) in hepatocytes infected with *P. cynomolgi* hypnozoites at 7 dpi. Note the association between hypnozoites and host mitochondria (**A**, **B**) or a host vacuole (**C**). PVM, parasitophorous vacuole membrane; PPM, parasite plasma membrane. Scale bars are 500 nm

IFA-CLEM was next employed to characterize the ultrastructure of liver stage hypnozoites (Figs. 4 and 5). Using the IFA protocol, UIS4 was successfully detected at the PVM of smaller liver stages, presumably hypnozoites (Fig. 4A). These hypnozoites exhibited 1–2 DNA-positive punctae (Fig. 4A) and could be located using CLEM (Fig. 4). Interestingly, hypnozoites were found to have a close association with host mitochondria (Figs. 4A-C, E, 5A, B and Additional File 6). In the surrounding of hypnozoites, host mitochondria often appeared damaged, swollen and/or showed abnormal internal membrane structures (Figs. 4A-C, E, 5A, B and Additional File 6). Large host vacuoles with heterogeneous content were also observed in association with hypnozoites (Fig. 5C). The apicoplast was in a defined area in all five hypnozoites (Figs. 4, 5D and E), in line with previous fluorescence microscopy data for *P. vivax* hypnozoites at a similar time point [23]. Additionally, the mitochondrial network (Fig. 5D), vacuoles (Fig. 5E) and up to one nucleus per hypnozoite (Figs. 4B, E, and 5F) were observed. A dense vacuole was detected in one hypnozoite (Fig. 5F). The nucleus was not identified for all hypnozoites potentially due to the difference in depth between nuclei localization and imaged sections. Interestingly, membrane-bound organized microtubule filaments were observed going through a part of one hypnozoite (Additional File 7). However, the significance and reproducibility of this observation need to be addressed with additional experimentations in the future. Overall, the results indicate that IFA-CLEM is a valuable tool for gaining insights into the biology of hypnozoites.

## Discussion

The approaches described in this paper utilize CLEM to characterize the ultrastructure of *Plasmodium* liver stages. The non-relapsing rodent parasite *P. berghei* was first used to optimize and compare CLEM protocols that rely on either genetically encoded GFP or an immunostaining assay to identify liver stages (referred to as GFP- and IFA-CLEM, respectively) (Figs. 1 and 2). The results revealed that, while most known parasite and host organelles were detectable using both protocols, certain organelles, such as the parasite ER, were more discernable using IFA-CLEM (Fig. 2 and Additional File 3). While IFA-CLEM caused more extraction and disruption of intracellular content compared to GFP-CLEM, ultrastructural features were generally well-preserved (Fig. 2). Based on these findings, it was concluded that IFA-CLEM could be a valuable tool to study the liver stages of *Plasmodium* species causing relapsing malaria, especially since they have low genetic tractability [2, 10] and are thus not well-suited for the use of genetically encoded tags. Accordingly, IFA-CLEM allowed us to assemble an unprecedented collection of images for the liver stages of *P. cynomolgi*, providing the first TEM micrographs of hypnozoites.

This study showcases the potential of utilizing CLEM to gain insights into the biology of *Plasmodium* parasites and their interactions with the host during the liver phase of infection. On one hand, a comprehensive and unbiased characterization of the ultrastructure of *Plasmodium* liver stages could lead to the discovery of new biological structures and processes. The observations of unknown *P. berghei* liver stage protuberances extending into the host cytosol (Additional file 4) and of the membrane-bound organized microtubule filaments within one *P. cynomolgi* hypnozoite (Additional file 7) exemplify this potential. In addition, comprehensive CLEM studies comparing different infection time-points have the potential to provide valuable insights into the intricate development of liver stage schizonts and hypnozoites. On the other hand, by adopting a more targeted imaging approach, CLEM could enhance the understanding of known biological processes that occur during liver stage development. These include the formation of the tubovesicular network (TVN) [13, 24], the dynamic changes in the parasite's organelle morphology and interaction [25] and the liver stage prominence, a thickening of the PVM of currently unknown nature and function [23]. Moreover, focusing CLEM studies on the surrounding of the PVM will facilitate a better understanding of the interactions between parasites and host organelles, which play a role in liver stage development [26–28].

The findings of this study confirm that the host mitochondrial network frequently localized near the PVM of liver stages (Figs. 3–5), as previously reported [26]. The proximity observed between host mitochondria and liver stage schizonts may be attributed to the spatial hindrance caused by mature schizonts occupying most of the space in infected hepatocytes. However, hepatocytes infected with hypnozoites offer ample space, and the association between host mitochondria and hypnozoites suggests that active interactions might be taking place. Host mitochondria play crucial roles in numerous cellular processes and their interactions with parasites might influence liver stage development by impacting nutrient uptake [26], host programmed cell death pathways [29], and immune responses [30, 31]. Interestingly, host mitochondria with abnormal morphologies were observed in the surrounding of hypnozoites (Figs. 4–5, Additional file 6) and have been previously associated with stress and pathological conditions [32–34]. This intriguing observation warrant further investigation using quantitative methods and suitable controls. However, as primaquine affects host mitochondria in addition to its anti-malarial activity [35, 36], it is tempting to speculate that primaquine’s effectiveness against hypnozoites is partially driven by its impact on the hepatocyte mitochondrial network.

An important limitation of this study is the lack of 3-dimensional analysis of samples. The features of liver stages and infected cells likely vary significantly across samples and certain structures might be only present at specific depths. To overcome this limitation, future research should focus on adapting volume EM approaches allowing to image samples at high resolution and in three dimensions [37, 38]. Especially, FIB-SEM shows promise for studying host–pathogen interfaces in three dimensions [39] and has already been used to characterize blood stages of *P. falciparum* [40]. Another limitation is the throughput of CLEM compared to other imaging methods, which makes it difficult to design studies with large numbers of conditions and to quantify any phenotypes with accuracy. Expansion microscopy, a method overcoming the diffraction limit of light microscopy by physically expanding samples, has a better throughput than CLEM and already provided insights into the biology of malaria [41, 42]. Even though expansion microscopy does not achieve the same resolution as EM, it is amenable to quantification and should be used alongside CLEM to obtain a better understanding of *Plasmodium* liver stages in the future.

## Conclusions

This study optimized CLEM to characterize the liver stages of *Plasmodium* parasites. Specifically, two approaches were evaluated: GFP-CLEM, which relies on parasites expressing GFP, and IFA-CLEM, which uses an immunofluorescence assay to accurately localize specific areas of interest. While IFA-CLEM caused more extraction of intracellular contents than GFP-CLEM, it generally preserved samples’ features and allowed for the identification of organelles. To further support that IFA-CLEM is a valuable tool to study *Plasmodium* species with low genetic tractability, experiments were performed with the liver stages of *P. cynomolgi* and provided first TEM micrographs of hypnozoites. The methods described in this paper could be used to better understand the liver stages of *Plasmodium* species, including *P. vivax*.

### Supplementary Information

**Additional file 1: **Portable Document Format (.pdf). Detailed protocols to perform GFP-CLEM and IFA-CLEM on *Plasmodium* liver stages. Supplemental protocols.

**Additional file 2: **Portable Document Format (.pdf). Samples used in this study. Supplemental table.

**Additional file 3: ***P.* *berghei* endoplasmic reticulum (ER) in Huh7 cells. Micrographs of *P. berghei* ER in Huh7 cells at 2 dpi imaged using GFP-CLEM (A) and IFA-CLEM (B). The same two micrographs are shown in Fig. 2D but were cropped differently. Scale bars are 1 μm. Supplemental figure.

**Additional file 4: **Unknown *P. berghei* liver stage protuberances extending in the host cytosol. Micrographs are from Huh7 cells at 2 dpi and were obtained with GFP-CLEM (A–B) and IFA-CLEM (C–D). Scale bars are 1 μm. Supplemental figure.

**Additional file 5: **Host mitochondria that localized in proximity to the PVM of *P. cynomolgi* liver stage schizonts. (A–D) Micrographs are from primary NHP hepatocytes infected with *P. cynomolgi *schizonts at 7 dpi and highlight host mitochondria (M_H_) in proximity to the parasitophorous vacuole membrane (PVM). PPM, parasite plasma membrane. Scale bars are 1 μm (A–C) or 500 nm (D). Supplemental figure.

**Additional file 6: **Mitochondria with abnormal morphologies that localized in proximity to the PVM of *P. cynomolgi* hypnozoites. (A–B) Micrographs are from primary NHP hepatocytes infected with *P. cynomolgi *hypnozoites at 7 dpi. Host mitochondria that clearly showed abnormal morphologies (e.g., swelling, signs of damage or abnormal internal membrane structures/vacuoles) are indicated with red asterisks. The micrograph in (A) is also shown in Fig. 4B but was cropped differently. PVM, parasitophorous vacuole membrane; PPM, parasite plasma membrane; N_*Pc*_, *P. cynomolgi* nucleus. Scale bars are 1 μm. Supplemental figure.

**Additional file 7: **A membrane-bound protrusion with organized microtubule filaments within a *P. cynomolgi* hypnozoite. (A–D) Micrographs are from primary NHP hepatocytes infected with a *P. cynomolgi* hypnozoite at 7 dpi. Micrographs (A–B) and (C–D) were taken at different depths. In (A), the object of interest is indicated with a red asterisk. PVM, parasitophorous vacuole membrane; PPM, parasite plasma membrane; N_*Pc*_, *P. cynomolgi* nucleus. Scale bars are 1 μm. Supplemental figure.

## Data Availability

Not applicable.

## Abbreviations

CLEM: Correlative light-electron microscopy
D-PBS: Dulbecco's phosphate-buffered saline
DPI: Day post-infection
EM: Electron microscopy
EN: Endomembrane network
ER: Endoplasmic reticulum
FIB-SEM: Focused ion beam scanning electron microscopy
G6PD: Glucose-6-phosphate dehydrogenase
GFP: Green fluorescent protein
GFP-CLEM: Correlative light-electron microscopy relying on a GFP-expressing organism
HSP70: Heat shock protein 70
IFA: Immunofluorescence assay
IFA-CLEM: Correlative light-electron microscopy relying on IFA
LM: Light microscopy
NHP: Non-human primates
PPM: Parasite plasma membrane
PV: Parasitophorous vacuole
PVM: Parasitophorous vacuole membrane
ROI: Region of interest
RT: Room temperature
TEM: Transmission electron microscopy
TVN: Tubovesicular network
UIS4: Upregulated in infectious sporozoites 4
