# Supplementary material for: Correlative light-electron microscopy methods to characterize the ultrastructural features of the replicative and dormant liver stages of Plasmodium parasites
Source: Malar J. 2024 Feb 21;23:53. doi: 10.1186/s12936-024-04862-w (PMC10882739; doi:10.1186/s12936-024-04862-w)
Supplement: Supplementary file 1 — Additional file 1: Portable Document Format (.pdf). Detailed protocols to perform GFP-CLEM and IFA-CLEM on Plasmodium liver stages. Supplemental protocols. [file 12936_2024_4862_MOESM1_ESM.pdf]

Infection of Huh7 cells with *Plasmodium berghei*

1. Grow Huh7 cells in Dulbecco's modified Eagle's medium (DMEM) with high glucose (Gibco) supplemented with 10% fetal bovine serum (FBS; Corning), 100 U/mL of penicillin-streptomycin (Gibco) and 1% GlutaMAX™ (Gibco) (hereafter referred to as Huh7 medium).

2. Coat dishes containing gridded coverslips (MatTek, P35G-1.5-14-CGRD) with poly-D-lysine (PDL):
    a. Add 2mL of 50 µg/mL of poly-D-lysine (Gibco) diluted in Dulbecco's Phosphate Buffered Saline (D-PBS; Gibco) in each dish and incubate for 1h at room temperature (RT)
    b. Wash 3 times with 2 mL of D-PBS
    c. Remove any traces of liquid and dry, with lids off, in a biosafety cabinet for 2h
    d. Use immediately or store at 4°C

3. Detach and seed cells (from step 1) using Trypsin-EDTA (0.25%; Gibco) at 700,000 cells per PDL-coated dish in 2.5 mL of Huh7 medium, one day prior to the infection.

4. Using microdissection, isolate sporozoites from the salivary glands of *Anopheles* mosquitoes infected with *P. berghei*, as previously described with modifications [1, 2]. Mosquitoes infected with *P. berghei* ANKA GFP-LUC$_{CON}$ [3] were produced by the SporoCore (University of Georgia) and used for this study.

5. Right before the infection, dilute sporozoites to desired concentration (e.g., 500,000 sporozoites per dish) in Roswell Park Memorial Institute (RPMI; Gibco) 1640 medium supplemented with 20% FBS.

6. Spin-infect Huh7 cells with 2mL of sporozoite suspension at 330 × g for 3 min, using low brake and acceleration settings.

7. Incubate cells for 2h at 37°C and 5% CO_2. Change the cell culture medium with fresh Huh7 medium and further incubate infected cells at 37°C and 5% CO_2 until the desired time point.

Infection of primary hepatocytes with *Plasmodium cynomolgi*

1. Coat dishes containing gridded coverslips (MatTek, P35G-1.5-14-CGRD) with collagen:
    a. Add 2mL of 0.02 N acetic acid containing 50 µg/mL of rat tail collagen I (Corning or Enzo) in each dish and incubate for 2h at RT
    b. Wash 3 times with 2 mL of D-PBS
    c. Remove any traces of liquid and dry, with lids off, in a biosafety cabinet for 2h
    d. Use immediately or store at 4ºC

2. Seed 2×10^6 primary non-human primate hepatocytes (BioIVT) per collagen-coated dish two days prior to the infection:
    a. Thaw and dilute hepatocytes 1/10 in pre-warmed INVITRO CP medium (BioIVT) supplemented with 1% of a Penicillin-Streptomycin-Neomycin (PSN) mixture (ThermoFisher) (hereafter referred to as 1% PSN Hep medium)
    b. Pellet hepatocytes at 100 × g for 5 min, using low brake and acceleration settings
    c. Resuspend hepatocytes at the desired concentration in 2.5 mL of 1% PSN Hep medium and incubate at 37ºC and 5% CO_2
    d. Next day and every other day, monitor the quality of cell monolayers using light microscopy

3. Using microdissection, isolate sporozoites from the salivary glands of *Anopheles* mosquitoes infected with *P. cynomolgi*, as previously described with modifications [1, 2, 4]. During this study, mosquitoes infected with the B strain of *P. cynomolgi* were produced by the Armed Forces Research Institute of Medical Sciences (AFRIMS; Bangkok, Thailand) under the IACUC approved animal use protocol PN22-10 "Production of *Plasmodium* spp. infected blood products utilizing a Rhesus Monkey (*Macaca mulatta*) malaria model".

4. Dilute sporozoite to desired concentration (e.g., 0.5-1 million sporozoites per dish) in 1% PSN Hep medium.

5. Spin-infect hepatocytes with 2 mL of sporozoite suspension at 200 × g for 5 min, using low brake and acceleration settings.

6. Incubate cells for 2h at 37ºC and 5% CO_2. Change cell culture medium with 2.5 mL of fresh 1% PSN Hep medium and further incubate infected cells at 37ºC and 5% CO_2.

7. Next day and every other day, change medium with 5% PSN Hep medium and further incubate infected cells at 37ºC and 5% CO_2 until the desired time point.

Light and fluorescent microscopy imaging for GFP-CLEM

1. Incubate cells for 30 min in FluoroBrite DMEM medium (Invitrogen) supplemented with 10% FBS, 100 U/mL of penicillin-streptomycin, 1% GlutaMAX^{TM} (hereafter referred to as Imaging medium) and 10ug/mL Hoechst (ThemoFisher) at 37ºC and 5% CO_2.

2. Wash cells once with Imaging medium.

3. Generate fiducial markers by scraping off linear patterns of cells from the monolayers attached to the gridded coverslips. This step is required since hepatocyte monolayers are too thick to allow the visualization of the gridded alpha-numerical labeling of the coverslips. Gentle scraping was conducted with a thin needle.

4. Change medium for fresh Imaging medium and perform imaging at RT using light and fluorescence microscopy. Limit imaging time or use a microscope with an environmental chamber. For each region of interest (ROI), acquire images at a range of magnifications (e.g., using 4×, 10×, 20× and 40× objectives) to facilitate the tracking of ROIs and the correlation between light and electron microscopy images.

5. Wash samples twice with D-PBS and incubate samples for 15 min at RT in D-PBS containing 2% paraformaldehyde (EMS) and 2% glutaraldehyde (EMS).

6. Keep samples at 4ºC, in the dark and in the fixative solution, until processing for CLEM.

Immunostaining and light and fluorescent microscopy imaging for IFA-CLEM

1. Incubate samples in D-PBS containing 4% paraformaldehyde for 15 min at RT and wash twice in D-PBS. Store samples at 4ºC in D-PBS, if needed.

2. Incubate samples in D-PBS containing 0.05% Triton X-100 (Sigma-Aldrich) for 15 min.

3. Wash samples twice in D-PBS and incubate samples for 30 min in D-PBS containing 2% bovine serum albumin (BSA; Sigma-Aldrich) at RT.

4. Incubate samples with primary antibodies for 3h in 2% BSA D-PBS. Primary antibodies used for this study were αUIS4$_{Pb}$ goat IgG (SICGEN; 1:250) or αUIS4$_{Pc}$ human IgG (1:2,000). The αUIS4$_{Pc}$ human IgG was produced in-house and is a mouse-derived antibody inserted into the human antibody backbone [2].

5. Wash samples 4 times in D-PBS and incubate with secondary antibodies for 90 min in 2% BSA D-PBS containing 2 µg/mL Hoechst at RT. Secondary antibodies used in this study were Alexa Fluor 568 donkey anti-goat IgG or Alexa Fluor 488 goat anti-human IgG (Invitrogen; 1:1,000).

6. Wash cells twice with D-PBS.

7. Generate fiducial markers by scraping off linear patterns of cells from the monolayers attached to the gridded coverslips. This step is required since hepatocyte monolayers are too thick to allow the visualization of the gridded alpha-numerical labeling of the coverslips. Gentle scraping was conducted with a thin needle. Wash cells twice with D-PBS.

8. Perform imaging at RT using light and fluorescence microscopy. For each ROI, acquire images at a range of magnifications (e.g., using 4×, 10×, 20× and 40× objectives) to facilitate the tracking of ROIs and the correlation between light and electron microscopy images.

9. Wash samples twice with D-PBS and incubate samples for 15min at RT in D-PBS containing 2% paraformaldehyde and 2% glutaraldehyde (EMS).

10. Keep samples at 4ºC, in the dark and in the fixative solution, until processing for CLEM.

Preparation of samples for TEM imaging

1. Rinse samples 3 times for 5 min each at RT in PBS, pH 7.4. During all steps, place the small MatTek plates inside a larger petri dish to reduce direct handling. Using a scribe pen, mark the side wall of each plate with identifying information.

2. Immerse samples in 1% osmium tetroxide and 1.6% potassium ferricyanide in PBS for 30 min. Keep covered from light, no agitation required.

3. Rinse samples in PBS 3 times and then once in distilled water for 5 min each at RT.

4. Subject samples to an ascending ethanol gradient (35%, 50%, 70%, 80%, 90%; 7 min, RT) followed by pure ethanol (3×; 5 min, RT).

5. Progressively infiltrate samples, while rocking, with normal hardness Epon resin (EMS) with BDMA accelerant. Infiltrate with 100% resin 3 times for 30-60 min each. Ensure that only a thin amount of resin remains within the glass bottom dishes to enable the best possible chance for separation of the glass coverslip. To do this, slowly pour off all resin that is possible to remove (do not pipette as this may introduce air bubbles). Add back fresh resin one drop at a time to the inner well of the MatTek dish until the surface of the well is just slightly filled below the layer of surrounding plastic. Place the dishes onto cardboard and then polymerize at 60ºC for 12-18 hours.

6. Remove glass coverslips using ultra-thin Personna razor blades (EMS) and liquid nitrogen (LN_2) exposure, as needed. To remove the coverslip, break the ultra-thin double razor into single blades by pinching the blade in half with the paper cover still on. While holding the MatTek dish upside down (i.e., coverslip side up), slide the single blade between one of the square edges of the coverslip and the plastic. The blade will need to be held with a very slight bend to both assist in applying gentle upward pressure and to ease the blade closer to the surface of the plastic dish (due to the ridge along the bottom of the MatTek plate). With small rocking motions, move the edge of the razor blade across the plastic plate and toward the opposite corner of the coverslip. As this happens, a small border of separation of the coverslip from the resin/plastic will be observed. Propagate that edge slowly but without halting. If the glass breaks, take care in removing the loose glass. Hold the dish coverslip side down and grab the edge of the plate with forceps while lowering the bottom of the dish (and the entire coverslipped region) into the LN_2 so that just the bottom of the plate is submerged. Avoid plunging the whole dish into LN_2. Once the crackling and popping noises cease, bring the plate back into position to proceed with the ultra-thin razor blade separation method described above. Repeat the LN_2 exposure and razor blade separation as needed until the entire coverslip has been removed.

7. Identify ROIs using the gridded alpha-numerical labeling and then mark the ROIs using a diamond or tungsten carbide scribe pen by drawing small boxes around the ROI on the

surface of the resin. It is easiest to do this with the aid of a dissection microscope and while the resin it still contained within the MatTek plate. Once the ROI have been marked, the exposed resin surface can be pushed out of the center of the MatTek dish by covering it with a tissue and pushing with a thumb. Take care not to scratch the surface of the resin as that can damage the cells beneath the resin surface.

8. Separate the marked ROIs with ultra-thin razor blades and with the disk of resin held on a glass slide covered with double-sided tape to keep the pieces of resin stable during cutting. To prepare blocks for sectioning, mount the individual ROIs onto labeled blank resin blocks using cyanoacrylate glue.

9. Cut serial thin sections (80-90 nm) using an ultramicrotome from the surface of the block (corresponding to the bottom of the cell layer) until approximately 20 microns in depth to ensure complete capture of the cell volumes. Sequentially collect section-ribbons onto formvar-coated slot grids. During the serial sectioning process, collect a few thicker sections (250-350 nm) onto glass slides. Stain these thicker sections with toluidine blue (T-blue) to track the cells of the ROIs and to serve as a bridge from the fluorescence to the TEM data.

10. Post-stain TEM grids with 2% uranyl acetate followed by Reynold's lead citrate, for 5 min each.

11. Image sections using 120kV TEM (FEI Tecnai 12 TEM) and cMOS camera (Gatan Rio16). During TEM, it is useful to have images taken of the T-blue stained thick sections and the fluorescence datasets for each ROI. The orientation of the sections on the TEM may have been flipped and/or rotated from the T-blue orientation. Identifying the schizont or hypnozoite structures within a specific cell requires a general mapping and correlation of the surrounding host cell nuclei, a process most easily done with the fluorescence, brightfield, and T-blue images. From that correlation, specific structures of interest can be quickly found via TEM. During TEM imaging, it is helpful to take a series of data covering from very low magnification (20-40 ×) to higher magnification (13,000 ×) to maintain the context from which the higher magnification images, particularly of hypnozoites, are collected. Lastly, when utilizing the serial sections for 3-dimensional context, keep track of the section order during TEM imaging.

12. Correlating images from CLEM datasets can be done with multiple approaches including freehand overlays with Adobe Photoshop or Microsoft PowerPoint. A more guided approach to overlaying can be followed as described by Keene et al. [5].

References

1. Vanderberg JP, Gwadz RW. The Transmission by Mosquitoes of Plasmodia in the Laboratory. In: Kreier JP, editor. Pathology, Vector Studies, and Culture: Academic Press; 1980. p. 153-234.
2. Maher SP, Bakowski MA, Vantaux A, Flannery EL, Andolina C, Gupta M, et al. A Drug Repurposing Approach Reveals Targetable Epigenetic Pathways in Plasmodium vivax Hypnozoites. bioRxiv. 2023.
3. Franke-Fayard B, Janse CJ, Cunha-Rodrigues M, Ramesar J, Buscher P, Que I, et al. Murine malaria parasite sequestration: CD36 is the major receptor, but cerebral pathology is unlinked to sequestration. Proc Natl Acad Sci U S A. 2005;102(32):11468-73.
4. Gupta DK, Diagana T. In vitro Cultivation and Visualization of Malaria Liver Stages in Primary Simian Hepatocytes. Bio Protoc. 2020;10(16):e3722.
5. Keene DR, Tufa SF, Wong MH, Smith NR, Sakai LY, Horton WA. Correlation of the same fields imaged in the TEM, confocal, LM, and microCT by image registration: from specimen preparation to displaying a final composite image. Methods Cell Biol. 2014;124:391-417.
