# Supplementary material for: Correlative light-electron microscopy methods to characterize the ultrastructural features of the replicative and dormant liver stages of Plasmodium parasites
Source: Malar J. 2024 Feb 21;23:53. doi: 10.1186/s12936-024-04862-w (PMC10882739; doi:10.1186/s12936-024-04862-w)
Supplement: Supplementary file 2 — Additional file 2: Portable Document Format (.pdf). Samples used in this study. Supplemental table. [file 12936_2024_4862_MOESM2_ESM.pdf]

Additional file 2. Samples analysed during this study[a]

CLEM approach	*Plasmodium* species	Sample ID (Dish – ROI)	# of LS selected[b]	# of LS located with CLEM[c]	Comments
GFP-CLEM	*P. berghei*	1-1	3 Sz	1 Sz	2 Sz not located
		1-2	3 Sz	0	Correlation unsuccessful
		2-1	16 Sz	12 Sz	4 Sz not located
		2-2	5 Sz	5 Sz	-
IFA-CLEM	*P. berghei*	3-1	4 Sz	3 Sz	1 Sz not located
		4-1	9 Sz	7 Sz	2 Sz not located
		4-2	4 Sz	4 Sz	-
IFA-CLEM	*P. cynomolgi*	5-1	1 Sz	1 Sz	-
		5-2	2 Hz	2 Hz	-
		5-3	1 Hz	0	Correlation unsuccessful
		5-4	1 Sz	1 Sz	-
		6-1	1 Sz	1 Sz	-
		6-2	1 Hz	1 Hz	-
		6-3	1 Hz	0	Correlation unsuccessful
		7-1	1 Sz, 1 Hz	0	ROI lost during processing
		7-2	3 Sz	3 Sz	-
		7-3	1 Hz	1 Hz	-
		8-1	1 Hz	1 Hz	-
		8-2	1 Sz, 1 Hz	0	ROI lost during processing
		8-3	1 Hz	1 Hz	-

[a] ID, identification; ROI, region of interest; LS, liver stage; Sz, schizont; Hz, hypnozoite.

[b] Number of liver stages mapped using light and fluorescence microscopy and selected for CLEM imaging.

[c] Number of liver stages located and imaged using CLEM.
