# Supplementary figures and images for: Correlative light-electron microscopy methods to characterize the ultrastructural features of the replicative and dormant liver stages of Plasmodium parasites
Source: Malar J. 2024 Feb 21;23:53. doi: 10.1186/s12936-024-04862-w (PMC10882739; doi:10.1186/s12936-024-04862-w)

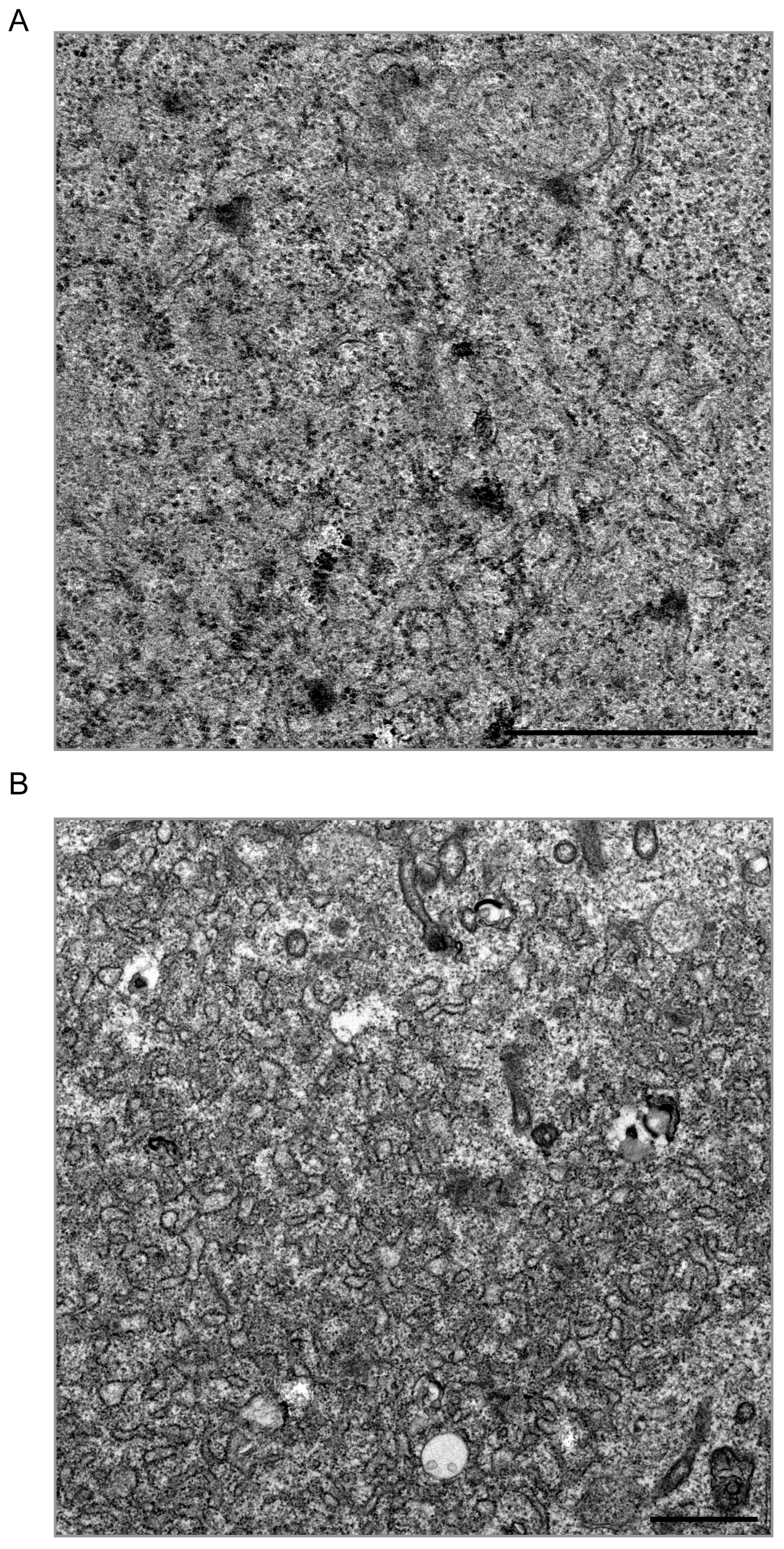

Supplement: Supplementary file 3 — Additional file 3: P. berghei endoplasmic reticulum (ER) in Huh7 cells. Micrographs of P. berghei ER in Huh7 cells at 2 dpi imaged using GFP-CLEM (A) and IFA-CLEM (B). The same two micrographs are shown in Fig. 2D but were cropped differently. Scale bars are 1 μm. Supplemental figure. [file 12936_2024_4862_MOESM3_ESM.tif]

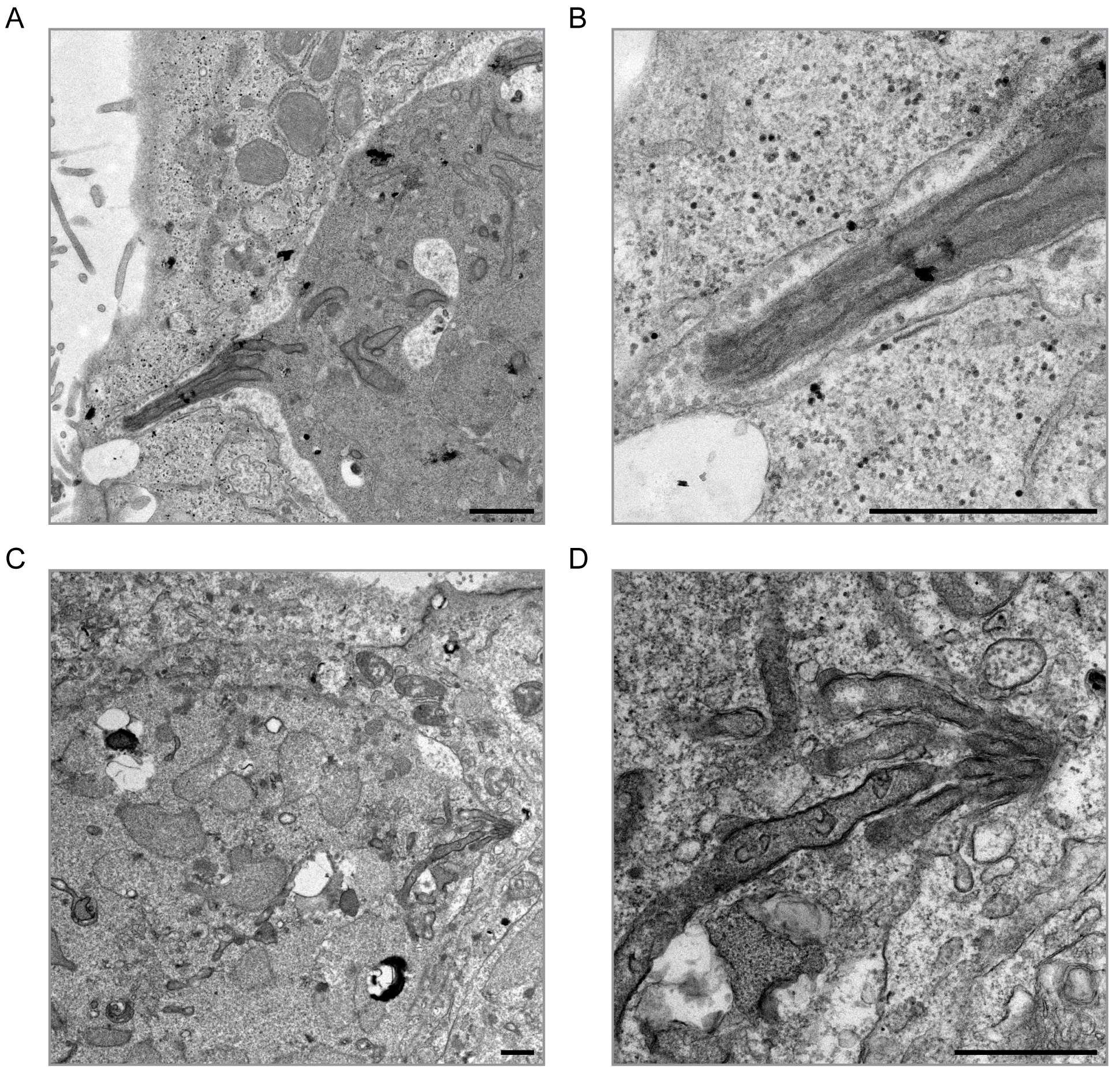

Supplement: Supplementary file 4 — Additional file 4: Unknown P. berghei liver stage protuberances extending in the host cytosol. Micrographs are from Huh7 cells at 2 dpi and were obtained with GFP-CLEM (A–B) and IFA-CLEM (C–D). Scale bars are 1 μm. Supplemental figure. [file 12936_2024_4862_MOESM4_ESM.tif]

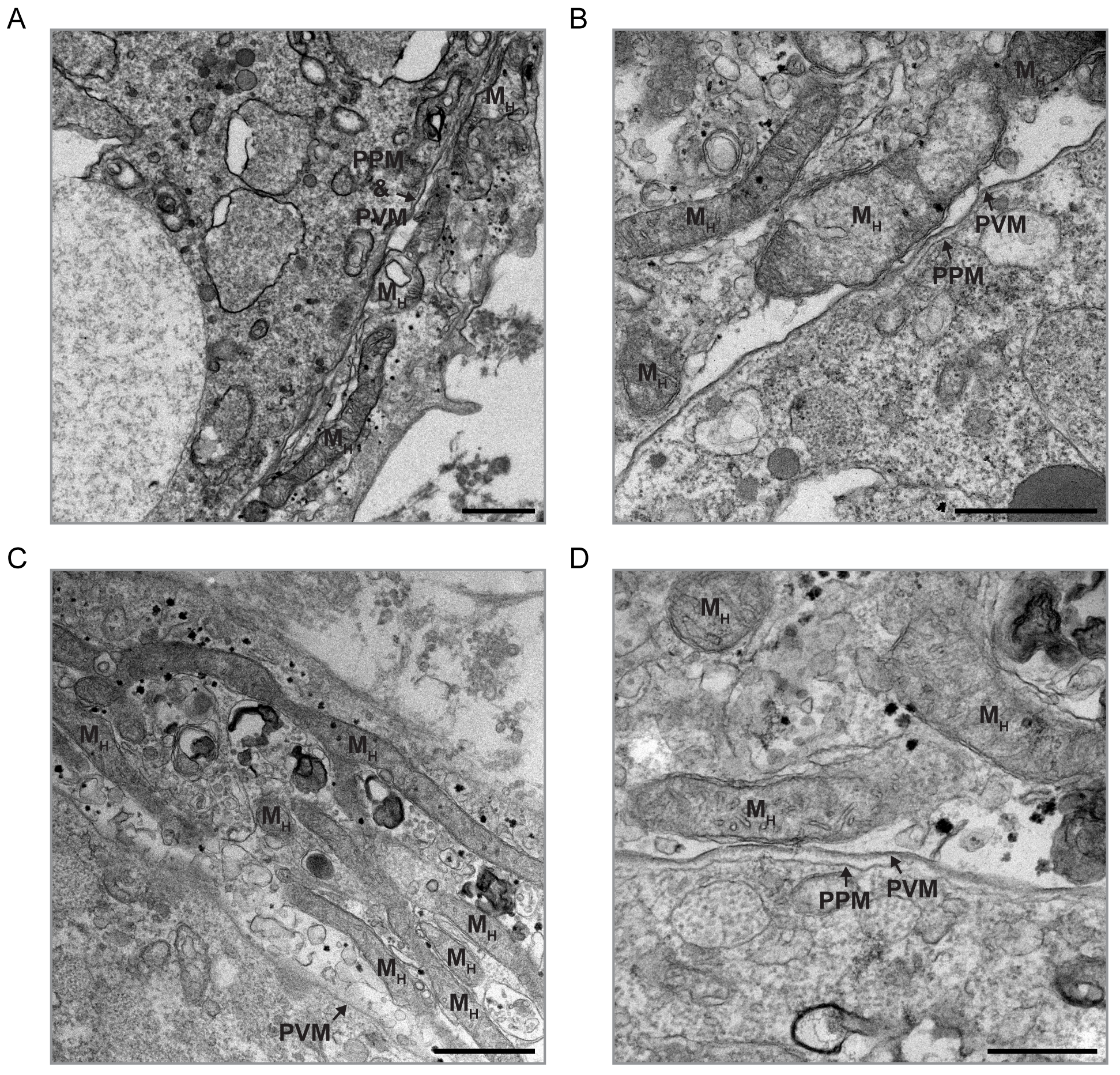

Supplement: Supplementary file 5 — Additional file 5: Host mitochondria that localized in proximity to the PVM of P. cynomolgi liver stage schizonts. (A–D) Micrographs are from primary NHP hepatocytes infected with P. cynomolgi schizonts at 7 dpi and highlight host mitochondria (MH) in proximity to the parasitophorous vacuole membrane (PVM). PPM, parasite plasma membrane. Scale bars are 1 μm (A–C) or 500 nm (D). Supplemental figure. [file 12936_2024_4862_MOESM5_ESM.tif]

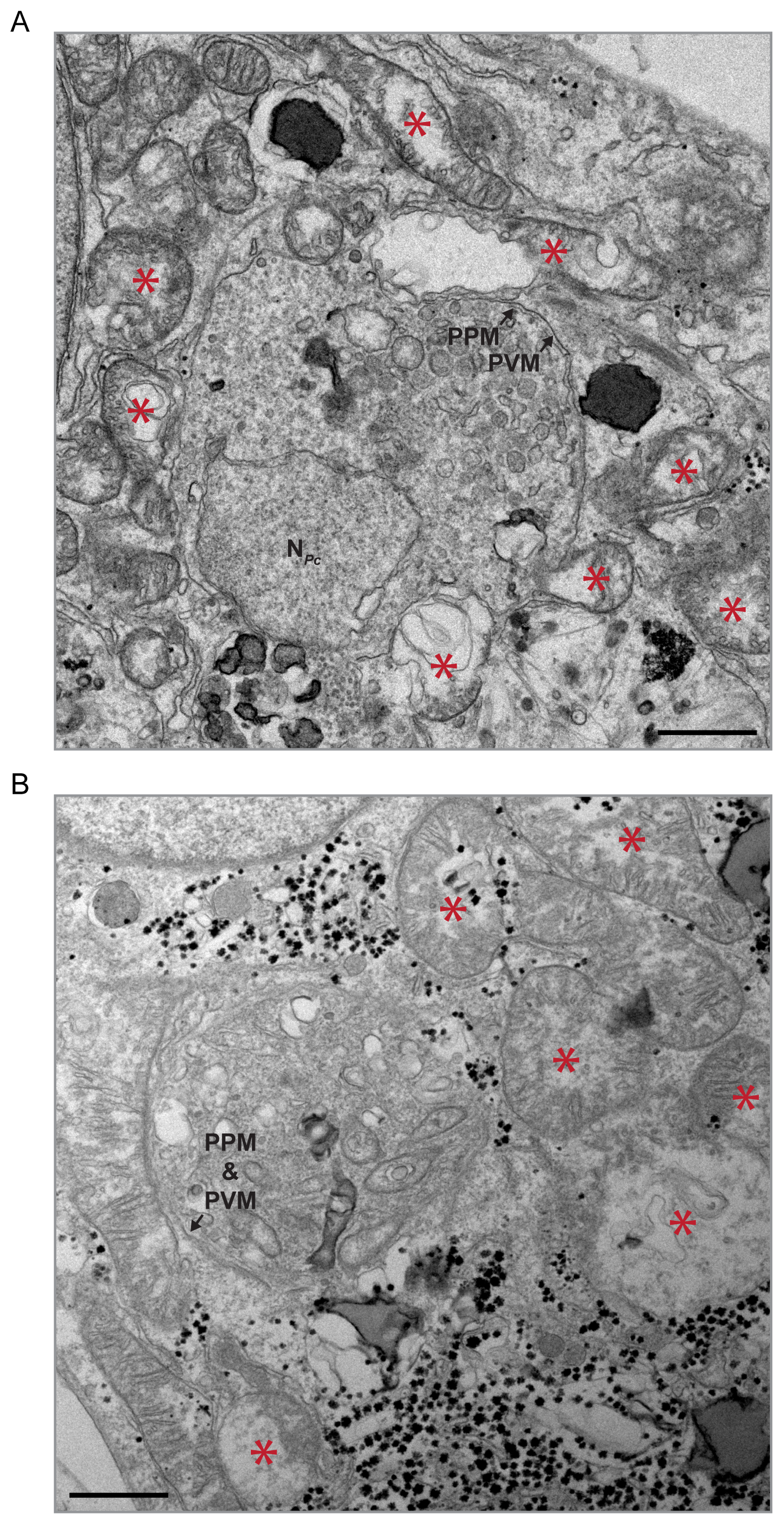

Supplement: Supplementary file 6 — Additional file 6: Mitochondria with abnormal morphologies that localized in proximity to the PVM of P. cynomolgi hypnozoites. (A–B) Micrographs are from primary NHP hepatocytes infected with P. cynomolgi hypnozoites at 7 dpi. Host mitochondria that clearly showed abnormal morphologies (e.g., swelling, signs of damage or abnormal internal membrane structures/vacuoles) are indicated with red asterisks. The micrograph in (A) is also shown in Fig. 4B but was cropped differently. PVM, parasitophorous vacuole membrane; PPM, parasite plasma membrane; NPc, P. cynomolgi nucleus. Scale bars are 1 μm. Supplemental figure. [file 12936_2024_4862_MOESM6_ESM.tif]

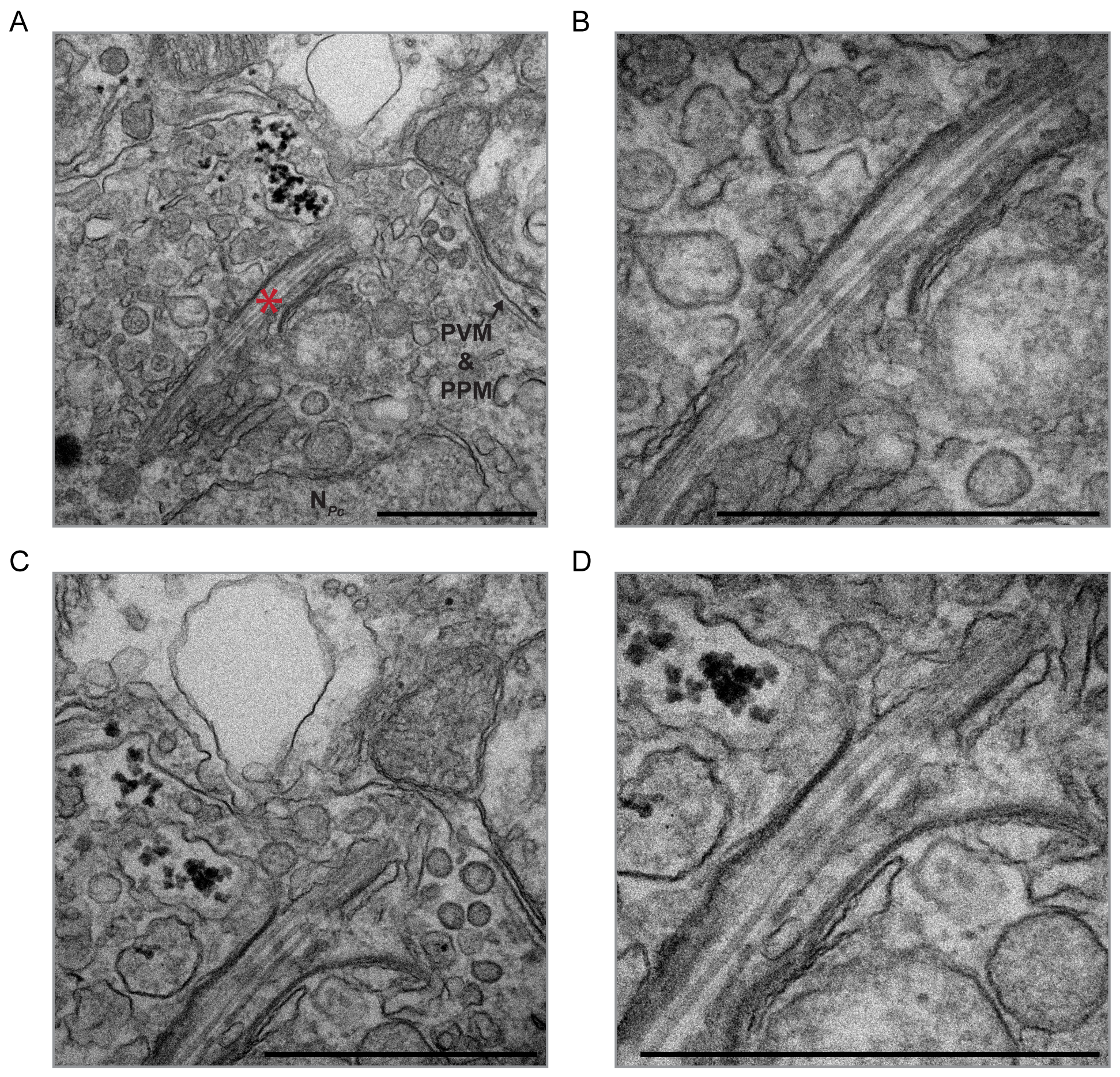

Supplement: Supplementary file 7 — Additional file 7: A membrane-bound protrusion with organized microtubule filaments within a P. cynomolgi hypnozoite. (A–D) Micrographs are from primary NHP hepatocytes infected with a P. cynomolgi hypnozoite at 7 dpi. Micrographs (A–B) and (C–D) were taken at different depths. In (A), the object of interest is indicated with a red asterisk. PVM, parasitophorous vacuole membrane; PPM, parasite plasma membrane; NPc, P. cynomolgi nucleus. Scale bars are 1 μm. Supplemental figure. [file 12936_2024_4862_MOESM7_ESM.tif]
